# RNA-Targeting Splicing Modifiers: Drug Development and Screening Assays

**DOI:** 10.3390/molecules26082263

**Published:** 2021-04-14

**Authors:** Zhichao Tang, Junxing Zhao, Zach J. Pearson, Zarko V. Boskovic, Jingxin Wang

**Affiliations:** Department of Medicinal Chemistry, University of Kansas, Lawrence, KS 66047, USA; zhichao.tang@ku.edu (Z.T.); zhao.junxing@ku.edu (J.Z.); zjp@ku.edu (Z.J.P.); zarko@ku.edu (Z.V.B.)

**Keywords:** alternative splicing, high-throughput screening, antisense oligonucleotide, small molecule, splicing modifier, RNA-targeting

## Abstract

RNA splicing is an essential step in producing mature messenger RNA (mRNA) and other RNA species. Harnessing RNA splicing modifiers as a new pharmacological modality is promising for the treatment of diseases caused by aberrant splicing. This drug modality can be used for infectious diseases by disrupting the splicing of essential pathogenic genes. Several antisense oligonucleotide splicing modifiers were approved by the U.S. Food and Drug Administration (FDA) for the treatment of spinal muscular atrophy (SMA) and Duchenne muscular dystrophy (DMD). Recently, a small-molecule splicing modifier, risdiplam, was also approved for the treatment of SMA, highlighting small molecules as important warheads in the arsenal for regulating RNA splicing. The cellular targets of these approved drugs are all mRNA precursors (pre-mRNAs) in human cells. The development of novel RNA-targeting splicing modifiers can not only expand the scope of drug targets to include many previously considered “undruggable” genes but also enrich the chemical-genetic toolbox for basic biomedical research. In this review, we summarized known splicing modifiers, screening methods for novel splicing modifiers, and the chemical space occupied by the small-molecule splicing modifiers.

## 1. Introduction

The splicing of messenger RNA precursors (pre-mRNAs) by the spliceosome is an essential processing step occurring before the translation of most nuclear encoded eukaryote genes. Some genes encoded in eukaryotic organelles (e.g., mitochondria [1]) and in prokaryotic cells [2] must undergo spliceosome-independent RNA splicing before translation. In the past two decades, gene- or exon-specific splicing modifiers have been developed for the treatment of several human disease states, including spinal muscular atrophy (SMA) [3,4], Duchenne muscular dystrophy (DMD) [5,6,7], and influenza virus infection [8], as well as pre-clinical drug development for diseases such as frontotemporal dementia and parkinsonism linked to chromosome 17 (FTDP-17) [9], yeast infection [10], and familial dysautonomia [11]. Instead of a traditional protein-targeting drug modality, these compounds act through direct binding to the pre-mRNAs.

### 1.1. Chemistry in RNA Splicing Reactions

**Figure 1:**
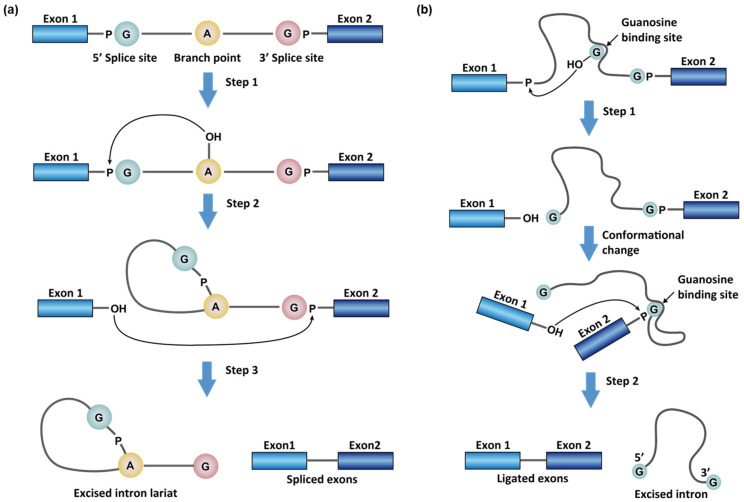
Splicing reaction with (**a**) adenosine as the branch point, which occurs in spliceosome-dependent splicing and group II introns, and (**b**) exogenous guanosine binding in the group I introns.

There are two general types of splicing reactions to process messenger RNAs (mRNAs)—the distinction being made based on the mechanisms of breaking the phosphate group in the 5′ splice sites (i.e., the exon/intron junction at the 5′ of the intron to be spliced). Both mechanisms start with a nucleophilic attack, but what distinguishes them is the identity of the nucleophiles. These can either be (a) an adenosine in the intron, namely the branch point, or (b) an exogenous guanosine cofactor that non-covalently binds to the intron aptamer [12] (Figure 1). The first type of splicing reaction occurs in the maturation of all the nuclear encoded mRNAs by spliceosomes (see ref [13,14] for recent reviews) and a family of intron sequences, namely group II introns, in bacteria, plants, and yeast [2]. This type of splicing reaction removes the intron as a cyclized fragment, intron lariat, in two steps (Figure 1a). First, the 2′-OH of the branching adenosine initiates nucleophilic attacks on the 5′ splice site and frees the 5′ exon (exon 1). Second, the 3′-OH of the 5′ exon attacks the 3′ phosphate group of the intron (3′ splice site) to rejoin the two exons with a simultaneous release of the intron lariat (Figure 1a). In animal cells, the intron lariats usually do not encode proteins and are commonly linearized and destroyed within minutes [15]. However, some of the intron lariats can remain cyclic and be exported to the cytoplasm to regulate cellular functions [15]. These cytosolic stable intron lariats are, therefore, a source of circular RNAs (circRNAs), whose regulatory role is not fully understood [16].

The second type of splicing involves a family of intron sequences: group I introns [12]. In this type of splicing reaction, a segment of the intron folds and forms a binding-pocket for exogenous guanosine (Figure 1b) [12]. The 3′-OH of the G then serves as a nucleophile and attacks the 5′ splice site. After the 5′ splice site breaks and is guanylated, the nucleophilic G is displaced by a G at the 3′ splice site in the binding pocket. The free 3′-OH of the 5′ exon subsequently attacks the 3′ splice site similarly to finalize the splicing reaction. In group I intron splicing, the intron product remains linear (Figure 1b).

In eukaryotes, most of the pre-mRNAs are spliced by spliceosomes. For this reason, we can also group the splicing reactions into two major categories, namely spliceosome-dependent and -independent splicing. These two types of splicing distribute distinctively in different kingdoms of life. Spliceosome, as alluded to already, only exists in eukaryotic cells and is enclosed in the cell nucleus. On the other hand, the spliceosome-independent group I and II introns are never observed in animal cells but they are broadly found in prokaryotic cells. Group I and II introns also exist in eukaryotic organelles, such as plant chloroplasts and yeast mitochondria. Other than messenger RNA (mRNA), transfer RNA (tRNA) [17] and ribosomal RNA (rRNA) [12] can also be spliced in a spliceosome-independent manner.

### 1.2. Spliceosome-Dependent Alternative Splicing

For nuclear encoded eukaryote genes, a single pre-mRNA sequence may have more than one splice pattern, leading to different protein isoforms from the same gene. The mechanism that generates these splice variants is termed alternative splicing. The alternative splicing of a particular pre-mRNA may happen in a single normal cell, different tissues or development stages, and some disease states (e.g., when a gene mutation is present). It is estimated that 90–95% of the human pre-mRNAs can undergo alternative splicing [18] (for reviews, see ref [19,20,21]).

**Figure 2:**
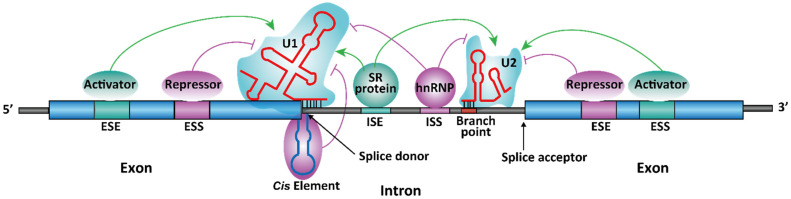
Regulatory mechanism for spliceosome-dependent splicing. ESE = exonic splicing enhancer, ESS = exonic splicing silencer, ISE = intronic splicing enhancer, ISS = intronic splicing silencer. A stem-loop structure at the 5′ splice was shown as an illustration of functional structural elements in regulating RNA splicing. The figure is modified from ViralZone, SIB Swiss Institute of Bioinformatics.

The process for choosing the spliced sites is highly regulated and is considered to be more complicated than the recognition of the primary intron sequences. Apart from the terminal and branch point sequences within the introns, other short RNA sequences in the pre-mRNA are also crucial in determining the splice sites and patterns. In general, there are four classes of such short sequences, namely exonic splicing enhancer (ESE), exonic splicing silencer (ESS), intronic splicing enhancer (ISE), and intronic splicing silencer (ISS) (Figure 2). These RNA sequences are also referred to as cis-acting regulatory sequence elements. The cis-acting elements exert their regulatory effect of splicing by binding to different RNA-binding proteins or noncoding (nc) RNAs [22], which function as splicing activators or repressors. These proteins and ncRNAs are also referred to as trans-acting regulatory elements, which directly or indirectly interact with the spliceosome subunits (Figure 2).

There are two large protein families of trans-acting elements in eukaryotic cells: heterogeneous nuclear (hn) RNP [23] and serine-arginine (SR) proteins [24]. The members of hnRNP family can be both splicing activators and repressors [23], although the most abundant proteins in this family, hnRNP A1 and A2, usually act as splicing repressors [23]. SR proteins, on the other hand, primarily function as splicing activators [24]. Some proteins, such as far upstream element-binding protein 1 (FUBP1), can exist as either a splicing activator or a repressor [25,26,27]. The regulatory role of such proteins depends on the context of the genes or the presence of the drug treatment [28].

Although pre-mRNAs are single-stranded (ss), stable secondary or tertiary structures form by intramolecular RNA folding. Some of these RNA structural elements are functional in regulating splicing (see Section 2.2 and Section 2.5 for examples). It is worth noting that it is still debated whether RNA secondary structures are commonly used to regulate RNA splicing. In an in vitro splicing system, it was demonstrated that only stem-loops with ~50 bp perfect match at the junction of the splices site can induce exon skipping [29].

### 1.3. Spliceosome-Independent Splicing

To date, three major types of spliceosome-independent splicing have been discovered for group I [30,31], group II [32,33], and tRNA introns [34,35]. Besides the three major types, the name “group III intron” was suggested for a rare splicing mechanism in mRNA genes of chloroplasts in euglenid protists [36,37], which has not been found elsewhere. The three major types of spliceosome-independent splicing are found in all three domains of life, but only tRNA splicing is observed in vertebrates [38]. The group I introns are self-cleaving ribozymes [39] and usually do not require a protein, although some RNA maturase can facilitate intron folding and promote splicing [40]. Figure 1b illustrates the mechanism of group I intron splicing. The group II intron splicing mechanism is similar to the spliceosome-dependent splicing (e.g., using an intronic A as a branch point, Figure 1a). The group II intron splicing machinery as a possible origin of the spliceosome in evolution has been suggested [41]. Both groups I and II introns are mostly found in bacteria, archaea, and chloroplast or mitochondria in plants and yeast [32]. They form complicated, functional, tertiary structures in RNA splicing [30,33], which can serve as druggable targets [10].

In plant chloroplasts and bacteria, the tRNA genes sometimes contain a group I or II intron and are spliced accordingly [34,42]. However, tRNAs are spliced via a completely different mechanism in eukaryotes and archaea [34,43]. This mechanism involves an endonuclease and a ligase to remove the introns and re-join the exons, respectively [38]. In mammalian cells, there are multiple copies of tRNA in the genome (e.g., more than 500 copies in human [44]), and 6% of the encoded tRNAs must be processed by tRNA splicing for their function [45]. The key differences between spliceosome-dependent and major types of spliceosome-independent splicing mechanisms are summarized in Table 1.

## 2. Known RNA-Targeting Splicing Modifiers for the Treatment of Human Diseases

Pharmacological modulation of RNA splicing may be used in two situations as a therapeutic modality for human diseases: (1) for diseases caused by mutations that induce aberrant RNA splicing, pre-mature stop codons, or reading frameshift. For the treatment of these diseases, correcting the mis-splicing or restoring the disrupted open reading frame by modulating splicing would be a promising strategy. Examples in this group include rare genetic diseases, such as DMD, SMA, and FTDP-17 (see Section 2.1, Section 2.2, and Section 2.5); (2) for diseases that are not caused by aberrant splicing, nonsense, or frameshift mutations, however, modulating splicing might lead to a reduced expression level of certain gene isoforms and ultimately mitigate the disease state. This category includes different infectious diseases (Section 2.3 and Section 2.4). The pharmacological approach targeting cis-acting regulatory elements in the pre-mRNAs has recently been validated as a strategy to alter the splice pattern in a gene-specific manner in cells, animal models, and patients.

There are two major classes of clinically validated splicing modifiers: (1) antisense oligonucleotides (ASOs) and (2) small molecules. ASOs are the current gold standard for modulating RNA splicing. In contrast to RNA interference (RNAi) that uses double-stranded (ds) RNAs to degrade the target pre-mRNAs through the RNA-induced silencing complex (RISC) [49], ASOs are ssDNA/RNAs that act by directly binding to the target pre-mRNAs through Watson-Crick base-pairing. Such stoichiometric binding to the cis-acting regulatory element prevents the pre-mRNA sequence from recruiting the splicing regulatory proteins or alters the equilibrium of the splicing-regulatory RNA structures. Therefore, the ASO binding to the splicing enhancer sequences (ESEs or ISEs) results in splicing inhibition, whereas ASO binding to the splicing silencer sequences (ESSs or ISSs) leads to splicing activation. Besides splicing modifiers, ASOs can also be used as a chemical-genetic tool to induce RNase H-dependent RNA degradation [50,51] or as a steric block [52,53,54] against the ribosome assembly.

Generally, the cellular uptake for ASOs is poor, which is the intrinsic limitation for their application [55]. Of particular interest is the inability of ASOs to cross the blood-brain barrier [55]. Illustrating this point is the FDA-approved ASO for the treatment of SMA, nusinersen, which must be administered by intrathecal injection into the cerebrospinal fluid to act on motor neurons in the central nervous system [3]. In addition, the immunogenicity of long ASOs is a liability for therapeutic uses and should be closely monitored in drug development [56]. On the other hand, cell-permeable RNA-targeting small molecules are an emerging pharmacological modality to modulate splicing. Risdiplam is the first small-molecule splicing modifier approved by the FDA for the treatment of SMA. Risdiplam readily crosses the blood-brain barrier and is formulated as an oral drug [4]. Risdiplam is also the first approved drug acting through binding to a non-ribosomal RNA. There are a number of excellent reviews on the topic of RNA-targeting small molecules in drug development (for recent reviews, see ref [57,58,59,60]). In this review, we only focused on the drug development of RNA-targeting splicing modifiers, including ASOs and small molecules (Table 2). RNA editing with CRISPR associated proteins (Cas) is another promising strategy that could potentially be used for modulating splicing. However, this review does not cover RNA editing due to lack of clinical validation and limitation of the scope (for recent reviews on RNA editing, see [61,62]).

### 2.1. Duchenne Muscular Dystrophy (DMD)

**Figure 3:**
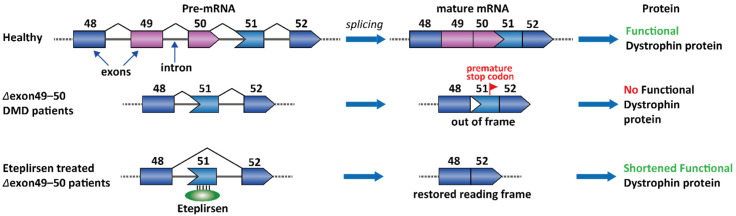
Therapeutic strategy of Eteplirsen for the treatment of DMD.

DMD is an X-linked recessive genetic disease. Certain gene mutations affect the expression of dystrophin protein, which is an essential component connecting the cytoskeleton of a muscle fiber to the surrounding extracellular matrix through the cell membrane [85]. DMD patients demonstrate muscle weakness from a young age and have a mean life expectancy of 19.0 years [86]. About 70% of patients with deletion mutations are amenable to partial dystrophin restoration by single exon skipping. For example, 14% of the DMD patients have a deletion in the genomic DNA sequence containing exons 49 and 50 [87]. Importantly, the nucleotide number in an exon is not necessarily an integer multiple of 3 for keeping a complete set of codons. The Δexon 49–50 transcript happens to cause a reading frameshift and creates a premature stop codon on exon 51. This frameshift and some other nonsense mutations in DMD patients can both create a premature stop codon, leading to nonsense-mediated decay of the transcript [88,89]. A clinically validated strategy is to skip the stop codon-containing exons in the transcript (exon 51) and restore the reading frame (Figure 3) [90]. Eteplirsen is the first FDA-approved ASOs for DMD. Eteplirsen binds to the ESE of exon 51 and induces exon skipping, which further leads to correction of the reading frame. Although the dystrophin is shorter than the wild-type protein, the Δexon 49–51 dystrophin is still partially functional [91].

Apart from the Δexon 49–51 genotype, many deletions cluster between exons 44 and 55, and therefore, this region has also been recognized as a “hotspot” target for ASO-based drugs. Similar to exon 51, exclusion of exons 53 and 45 would also result in partially functional dystrophin in 8% and 8% of the DMD patients, respectively [92]. Two ASOs, golodirsen and viltolarsen were discovered and approved for the treatment of DMD in patients with a confirmed deletion of the dystrophin gene that is amenable to exon 53 skipping [7,93]. Casimersen is the fourth approved ASO drug for DMD patients who have a confirmed mutation of the dystrophin gene that is amenable to exon 45 skipping [94]. DS-5141 is another ASO that induces exon 45 skipping and has completed the Phase I/II clinical trial (NCT02667483) [74].

### 2.2. Spinal Muscular Atrophy

**Figure 4:**
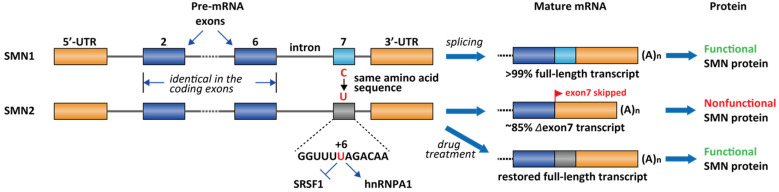
Differential splicing patterns for SMN1 and SMN2. ~85% of the SMN2 transcript has exon 7 skipped due to the C-to-T transition, which mediates the loss of interaction of SRSF1 and gain of the interaction of hnRNPA1. Most type I SMA patients have deleted SMN1 but wildtype SMN2. Nusinersen, RG-7916, and LMI-070 are all interventional therapy to restore exon 7 inclusion in SMN2.

**Figure 5:**
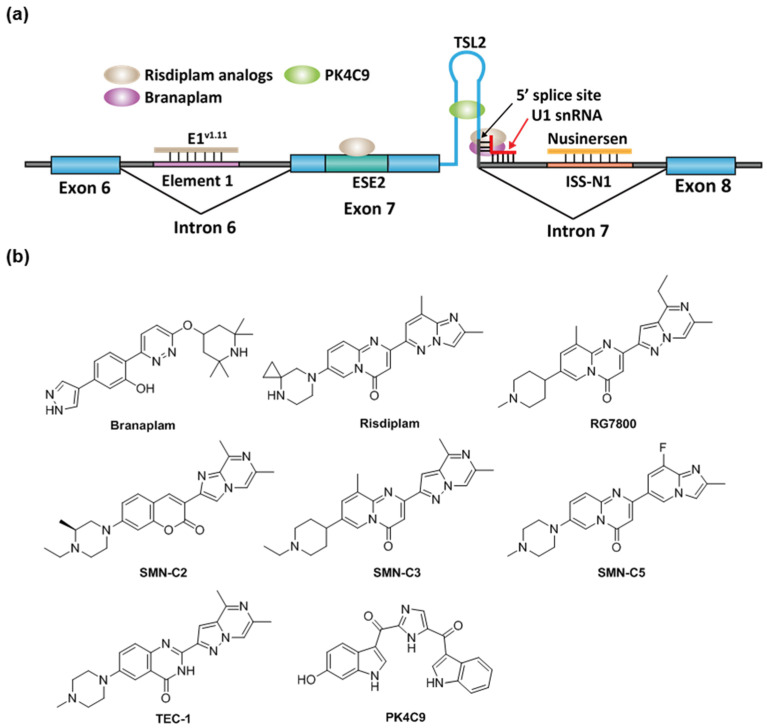
(**a**) Binding sites of the existing drugs for the treatment of SMA. Risdiplam has two binding sites on the SMN2 pre-mRNA exon7. (**b**) The structures of known RNA-targeting small-molecule splicing modifiers for SMN2 exon 7.

Spinal muscular atrophy (SMA) is one of the most common lethal genetic diseases in newborns [95]. The cause of SMA in the most severe type (type I) is a recessive homozygous deletion within the survival of motor neuron 1 (SMN1) gene in chromosome 5 [95]. Humans have two nearly identical genes, SMN1 and SMN2. However, the protein produced by SMN2 cannot fully compensate for the loss of SMN1 in type I SMA patients. It was demonstrated that a single nucleotide difference (C-to-T change at exon 7 at +6) in exon 7 of SMN2 causes ~85% of the exon 7 skipping [96], leading to an inactive SMN isoform (Δexon 7, Figure 4). The SMN2 exon 7 contains a regulatory RNA secondary structure at the 3′-end, namely terminal stem-loop 2 (TSL2) (Figure 5) [97]. Reverse genetic studies showed that TSL2 is inhibitory [97]. Destabilizing TSL2 by point-mutations leads to exon 7 inclusion. On the contrary, strengthen TSL2 causes exon 7 skipping even in SMN1 [97], probably because the formation of TSL2 partially competes with the binding of U1 snRNP.

The exact mechanism of SMN in motor neuron maintenance and survival is not fully elucidated. To date, there are a collection of drugs used in clinics or in the development pipelines for the treatment of SMA (for recent reviews, see refs [98,99]). Homozygous deletion of both SMN1 and SMN2 is embryonic lethal. Therefore, type I patients usually have a wildtype SMN2^+/+^ gene. One of the therapeutic strategies to cure SMA is to restore full-length splicing of SMN2 with a pharmacological intervention (Figure 4).

The current FDA-approved ASO, nusinersen, was obtained by chemical modification of ASO-10-27 (masking intron 7 +10 to +27, which covers the ISS-N1 region). ISS-N1 is a 15 nucleotide (intron 7 +10 to +24) inhibitory cis-element located downstream of the 5′ splicing site of exon 7 and was found to play a dominant role in inducing exon 7 skipping [100]. Several splicing factors were identified to bind ISS-N1, including hnRNPA1/A2 and SRSF10 [101,102]. It is likely the effect of nusinersen comes from sequestration of these factors from binding to ISS-N1. Following intrathecal injections, nusinersen significantly improves motor function in these SMA patients and restores the SMN level in the central nervous system and peripheral tissues, leading to a significant improvement of the survival rate [103]. Besides ISS-N1 in the intron 7, an ASO targeting the ISS in the intron 6, E1 region, is also efficacious in human cells and mouse models (Figure 5) [104]. Similar to nusinersen, ASO E1^v1.11^ prevents E1 region from recruiting splicing repressor proteins and thus rescues exon 7 inclusion [104]. RG-7800 is the first-in-class small-molecule splicing modifier (PTC/ Roche) tested in a clinical trial for the treatment of SMA. RG-7800 increases the production of full-length SMN2 mRNA upon oral administration in mouse models [105]. The clinical trial was stopped as a precautionary measurement for a retinal toxicity issue observed in cynomolgus monkeys after chronic daily oral dosing for 39 weeks [105]. Risdiplam (RG-7916) is a close analog of RG-7800 and is a second-generation molecule with enhanced potency and improved pharmacokinetics and safety properties [65]. Risdiplam is approved by the FDA for the treatment of patients in all stages of SMA in August 2020 [106]. Branaplam (Novartis, Cambridge, MA, USA) is another small-molecule splicing modifier and was tested in a Phase 2/3 clinical trial for the treatment of SMA. In the SMNΔ7 mouse model, branaplam treatment increased full-length SMN mRNA and protein levels, and extended survival [66]. The clinical trial was terminated after risdiplam was developed to a more advanced stage.

Risdiplam and branaplam have partially overlapping mechanism of action. They act by stabilizing the transient double-strand RNA structure formed by the SMN2 pre-mRNA and U1 snRNP complex [107,108]. NMR studies demonstrated that a risdiplam analog, SMN-C5, binds to a bulged A between the 5′ splice site and U1 snRNA and enhances the spliceosome recognition (Figure 5) [107]. However, the chromatography study demonstrated that the in vitro binding between branaplam and 5′ splice site requires holo U1 snRNP, rather than U1 snRNA alone [108]. The exact binding nucleotides for branaplam have not been elucidated. Compared to branaplam, risdiplam analogs not only bind to the interface between 5′ splice site and U1 snRNA, but also to a secondary binding site near the 3′ of the exon. RNA immunoprecipitation demonstrated that risdiplam analogs preferentially bind to a GA-rich sequence that matched the sequence of exon 7 +22 to +30 [28]. The analysis of other risdiplam-sensitive genes in splicing demonstrated that the GA-rich sequence was slightly enriched at the 3′ splice site but does not have a strong consensus in all sequences [109,110]. PK4C9 is another small molecule reported as an SMN2 splicing modifier (Figure 5b). It was identified by a target-based screening against TSL2 in exon 7. In SMA patient fibroblast cells, PK4C9 increased exon 7 inclusion by 40% at 40 μM, coupled with a 1.5-fold increase in SMN protein level. Reverse genetics demonstrated that the 5′ splice site (GAGUAAGU) of exon 7 is likely to be the target of PK4C9. NMR and molecular dynamics studies indicate that PK4C9 may improve the accessibility of the 5′ splice site via binding to TSL2 and stabilizing a tri-loop structure of TSL2 [67].

### 2.3. Virus Infection

Viruses can adopt different strategies to maximize the use of their DNA or RNA genomes and encode more than one peptide with a single nucleic acid sequence. Such strategies include RNA editing (in Ebola virus [111]), programmed frameshift (in coronavirus [112]), and alternative splicing (in influenza A virus [113]). Viruses hijack the host spliceosome to process its transcript, and this process may be assisted by some viral proteins.

Influenza A virus (IAV), a highly infectious and unpredictable respiratory pathogen, represents a substantial threat to public health [114]. The outbreak of Spanish flu (H1N1) in 1918 killed 21–50 million people globally [115], one of the deadliest pandemics in recorded human history. According to the United States Centers for Disease Control and Prevention (CDC), there have been 9.3–45 million cases of seasonal flu every year in the U.S. from 2010 to 2020, with 12,000–61,000 flu-associated deaths annually [116] (access date 11 April 2021). Two dominant influenza A strains (H1N1, H3N2) are currently circulating in the United States and globally [117] (access date 11 April 2021). Both influenza A and B are ssRNA(–) viruses (“–“ indicates the RNA sequence is reversely complemented to the coding sequence), and their genome contains eight RNA segments.

**Figure 6:**
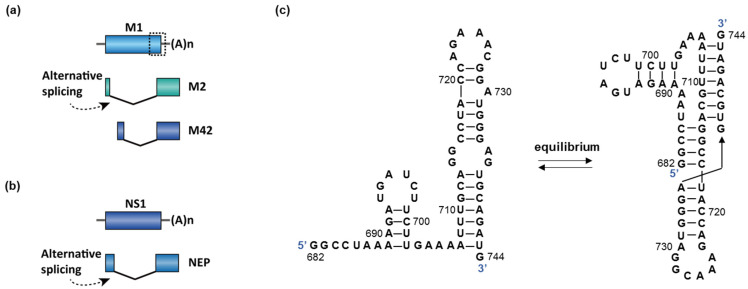
(**a**,**b**) Two genes in influenza A undergo splicing: M and NS1. The dotted box in M is the proposed region responsible for splicing switch (enlarged in (**c**)). The spliced isoform of NS1 is named nuclear export protein (NEP). (**c**) An equilibrium between a hairpin and a pseudoknot structure was proposed to control the splicing of M in influenza A [115].

RNA splicing occurs in segments 7 and 8, encoding matrix protein (M) and nonstructural protein (NS1), respectively (Figure 6a,b) [118]. The splicing of M is essential because the splice variants M1 (matrix protein) and M2/M42 (ion channel) are both required for viral replication. The M1 matrix protein assembles to form virus-like particles, which are a prerequisite for the budding process. M2 forms an ion channel on the nucleocapsid envelop and is essential for viral entry. Mutually exclusive secondary structures at the 3′ splice site of the M gene are proposed to control the splice pattern [115,119]. Specifically, the hairpin conformation promotes splicing of M and production of M2 (Figure 6c). In contrast, the pseudoknot conformation disrupts binding of the trans-acting factor SRSF1 to an exonic splice enhancer (ESE) and blocks the splice site (Figure 6c), and therefore favors the production of M1 [115,120]. However, the hairpin or the pseudoknot structures were not discovered by whole-genome chemical probing analyses in virus-infected cells [121,122]. A recent study demonstrated that IAV strains that are adapted in avian and human host cells contain different splicing regulatory RNA structural elements at the 3′ splice site of M [113]. The inhibition of NS1 also reduces the viral replication in vitro [123]. Influenza A virus hijacks the host spliceosome by the interaction with U2 and U6 snRNA through the viral protein NS1 [118].

ASOs have been developed to target the splicing of M1/M2. Radavirsen (AVI-7100) is the most advanced drug candidate that showed good safety and tolerability profile in a Phase I clinical trial [8]. Radavirsen is a 20-mer ASO targeting a conserved region that controls M1/M2 splicing. The sequence of radavirsen and experimental data for the in vitro drug effect has not been published.

**Figure 7:**
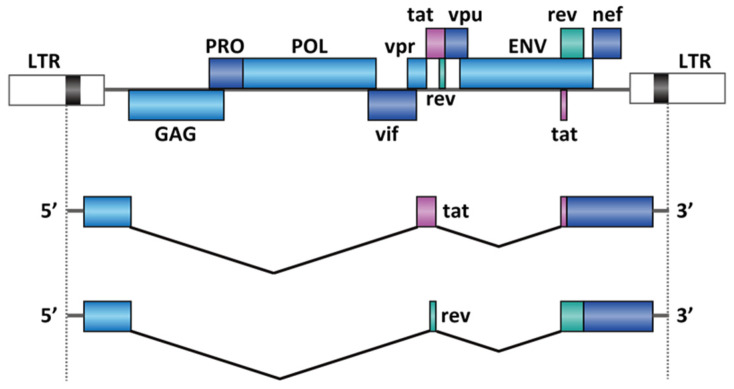
RNA splicing in HIV-1 genes tat and rev. The figure represents only two sets of the potential splice sites out of four alternative 5′ splice sites and eight alternative 3′ splice sites [125]. The figure is modified from ViralZone, SIB Swiss Institute of Bioinformatics.

Another notable example of viral splicing is in human immunodeficiency virus 1 (HIV-1). HIV-1 is an ssRNA(+) retrovirus. Unlike IAV, all the viral genes are encoded on a single strand of RNA. HIV is the cause of the acquired immunodeficiency syndrome (AIDS). In 2018, 37.9 million people were living with the virus in the world, and 770,000 people died from AIDS-related disease [124]. The ~9000 nucleotide HIV-1 genome transcribes over 50 functional mRNAs by using alternative splicing [125], including several essential genes, such as tat and rev [126]. There are two introns to be spliced in tat and rev (Figure 7), and the splicing of the 5′ intron is a prerequisite to 3′ intron splicing [126]. The pre-mRNA is differentially spliced to produce distinct mRNAs coding either for the tat or rev proteins (Figure 7). The protein Rev controls the export of unspliced or partially spliced transcript from the nucleus, which is crucial for viral replication and packaging [127,128]. Although earlier work demonstrated that splicing-switching ASOs reduced the release of HIV virions in cells [129], to our knowledge, no splicing modifier has shown promising in vivo activities for the treatment of HIV infection.

Apart from IAV and HIV, RNA splicing is also essential for some other viruses, such as human T-lymphotropic virus, parvovirus B19, and human papillomavirus 1. The genes that undergo RNA splicing for these viruses are summarized in Table 3.

### 2.4. Yeast Infection

Antifungal drugs against fungal infections especially against invasive fungal infections remain an unmet clinical need because of the unsatisfactory treatment outcomes with currently available antifungal drugs. One key fundamental challenge in new antifungal drug discovery is that fungi and yeast cells have similar biochemical pathways to humans [133]. Group II introns are large autocatalytic RNA motifs that adopt complex tertiary structures and catalyze RNA splicing [134]. They are found in plants, fungi, yeast, and various lower eukaryotes, but are not present in mammals [135]. Due to their absence in mammals and essential roles in fungal metabolism, group II introns may serve as potential targets for highly specific antifungal drug discovery.

Recently, the Pyle group reported a series of antifungal agents with novel structures that target yeast group II introns by inhibiting its ribozyme activity [10]. They identified two compounds termed intronistat A and intronistat B which were shown to inhibit group II intron splicing in *S. cerevisiae* in vivo and consequently cause its growth inhibition. They were also shown to selectively bind group II intron tertiary structure without affecting the other two known splicing systems (intron I and spliceosome). Importantly, intronistat A and intronistat B showed little toxicity in human cells, indicating specificity for fungi.

### 2.5. Frontotemporal Dementia and Parkinsonism Linked to Chromosome 17 (FTDP-17)

FTDP-17 is an autosomal dominant neurodegenerative disorder with symptoms of behavioral changes and cognitive and motor impairment [136]. The disease is primarily caused by mutations in the microtubule-associated protein tau (MAPT) gene [137]. The tau protein stabilizes neuronal microtubules under normal conditions. More than 38 mutations were identified in FTDP-17 patients, and the majority of the patients have a distorted proportion of tau isoforms [136]. Some mutations (e.g., c.892A > G [138], intron 10 +16C > T [139]) promote an exon 10 inclusion splice isoform, namely 4-repeat (4R), which leads to tau hyperphosphorylation and aggregation, and ultimately pathogenic neurofibrillary tangles [140,141].

Molecules that inhibit tau phosphorylation and aggregation, anti-tau antibodies, and ASOs targeting MAPT splicing and expression have been developed for the treatment of FTDP-17 (for a recent review, see ref [142]). It was shown that some ASOs that induce exon skipping also causes the reduction of expression level of the pathogenic tau, probably through nonsense-mediated decay pathway [75].

**Figure 8:**
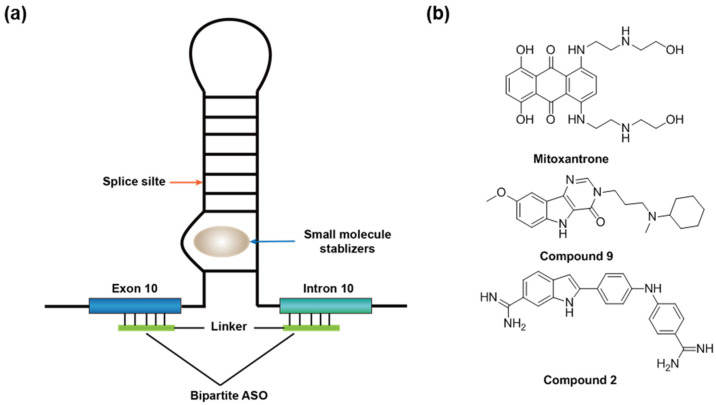
(**a**) A stem-loop controls the exon 10 splicing in the MAPT gene and the binding site of the known splicing modifiers. (**b**) The structures of known RNA-targeting small-molecule splicing modifiers for MAPT exon 10.

Besides ASO-based tau expression inhibitors, drug molecules that induce the exon 10 skipping in the mutant MAPT would also be of potential therapeutic interest for restoring the aberrant splice pattern for FTDP-17 [9]. ASOs bound to either the 5′ or 3′ splice site of exon 10 were demonstrated to induce exon 10 skipping [76]. An RNA stem-loop structure in intron 10 near the 5′ splice site acts as a splicing silencer (Figure 8a). This stem-loop structure can be destabilized via pathogenic mutations in the 5′ splice sites of exon 10 [143]. Therefore, stabilizing the stem-loop structure is a strategy to induce exon 10 skipping and restore the non-pathogenic MAPT isoform. An anticancer drug, mitoxantrone, was identified in a high-throughput screening campaign as the stem-loop structure ligand and inhibitor of exon 10 splicing (Figure 8b) [144]. NMR experiments demonstrated that mitoxantrone stabilizes the stem-loop structure through intercalating into the stem base [145]. Several other small-molecule splicing modifiers were identified by the Disney group via structure-based design [9,79] or phenotypic screening (e.g., “compound 9” and “compound 2” from the original report [9] (Figure 8b). Compound 9 was shown to stabilize an internal bulge of the stem-loop structure and reduce exon 10 splicing [9]. The Wolfe group also demonstrated that a bipartite ASO that is complementary to the flanking arms of the stem-loop can destabilize the structure and, thereby, inhibit the exon 10 splicing (Figure 8a) [77]. Importantly, a conjugate of this bipartite ASO and mitoxantrone is more potent than the ASO or mitoxantrone alone in the in vitro splicing reactions [78]. To our knowledge, this small molecule–ASO conjugate is the first small molecule–ASO chimera to modulate RNA splicing.

### 2.6. Familial Dysautonomia

Familial dysautonomia (FD) is an inherited autosomal recessive disease which occurs almost exclusively among the Ashkenazi Jewish with a carrier frequency of about 1 in 30 [146]. Features of this disease include loss of pain and temperature sensation, gastrointestinal dysfunction, respiratory abnormality, autonomic crises, progressive optic atrophy and gait ataxia [147]. FD was caused by mutations in the gene IKBKAP which encodes IKK complex-associated protein (IKAP)/elongator protein 1 (ELP1) [148,149]. More than 99% cases were found to be related with a T to C mutation at the donor splice site of intron 20 (intron 20 +6T > C). This mutation causes inefficient use of intron 20 donor splice site and results in skipping of exon 20 in the mature IKBKAP mRNA. Skipping of exon 20 generates a frameshift and consequently introduces a stop codon in the reading frame of exon 21, eventually generates truncated IKAP protein [147].

There is currently no targeted therapy for FD, but efforts have been made to correct exon 20 skipping and restore the full length IKAP protein expression. Based on their experience in discovery of ASO treatments for SMA, the Krainer group used a two-step screening approach to identify cis-elements in IKBKAP pre-mRNA and ASOs that could restore exon 20 splicing in FD patient fibroblasts [11]. Two splicing silencer elements were identified separately in intron 20 and intron 19. One ASO that masks intron 20 +7 to +26 nucleotides was found to be most effective and induced completely inclusion of exon 20 in IKBKAP transcript and statistically significant increase in IKAP protein levels in FD patient fibroblast cells [11]. In vivo effect was also validated in a mouse model with ASO 7-26S which contains a more stable phosphorothioate backbone in structure. Similarly, the Andresen group also identified a splicing silencer in intron 20 which acts as the binding site of hnRNP A1, one splicing repressor [80]. Their most active ASO candidate, SSO1 which masks intron 20 +11 to +35 nucleotides was able to induce completely restoration of IKBKAP exon 20 inclusion and dramatic increase in IKAP protein levels in FD patient fibroblast cells at low nanomolar concentration.

The Pagani group described a novel strategy to correct exon 20 skipping by using exon-specific U1 snRNAs (ExSpeU1s) [81]. ExSpeU1s are snRNA like particles that complementarily bind to intronic regions downstream of the 5′ splice site of skipped exons and facilitate exon recognition by recruiting the spliceosomal components, thus increase exon inclusion during splicing. Lentiviral transduction of FD fibroblasts with the most potent ExSpeU1s increased full length IKBKAP mRNA by three-fold and restores IKAP protein level to ~80% of the normal fibroblasts. In a TgFD9 transgenic mouse model, intraperitoneal delivery of ExSpeU1s adeno-associated virus particles successfully increased the production of full-length human IKBKAP transcript and protein [81].

Small molecule splicing modifiers were also reported to be able to correct exon 20 skipping and induce full length IKAP protein expression. Kinetin, a plant cytokinin used as anti-aging skin care agent, was found to dramatically increase exon 20 inclusion and consequent wild type IKBKAP mRNA amount as well as IKAP protein in FD cell lines [150]. Mechanism study revealed that this effect is independent of FD mutation while a motif containing CCA element at the joint of 3′ end of exon 20 and 5′ splice site was determined to be necessary for kinetin’s activity [151]. Kinetin was the first small molecule splicing modifier entered clinical trial. However, the trial was discontinued because of withdrawal of participants [82]. A kinetin analog, termed RECTAS, was identified as a more potent splicing modifier than kinetin in promoting exon 20 inclusion and IKAP expression in FD patient cells [83].

## 3. Screening Methods for RNA Splicing Modifiers

The deciding process for splice site selection can be influenced by many regulatory factors. For example, more than 46 different proteins were discovered as regulatory factors for the exon 7 inclusion/skipping in the SMN2 gene [152]. Due to this complexity, phenotypic screening is a widely used approach to identify novel splicing modifiers. as Among these, “ASO walking” for ASOs [101,153] and cell-based phenotypic assays for small molecules [10,89,154] find broadest use. Both clinically tested small molecules, branaplam and risdiplam, were originally uncovered by phenotypic screening campaigns [108,155]. Target-based rational design for small-molecule splicing modifiers is also emerging and were elicited for MAPT splicing via docking the MAPT exon 10 regulatory stem-loop and a collection of RNA-binding molecules [9]. In this review, we will focus on the approaches for the discovery of RNA-targeting splicing modifiers.

### 3.1. “ASO Walking” Uncovers Cis-Acting Factors

**Figure 9:**
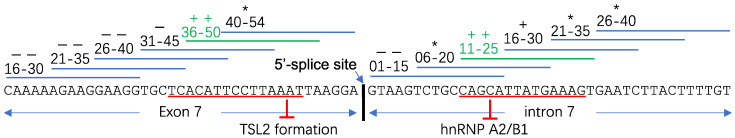
Binding sites for the ASOs used in the SMN2 exon 7 and intron 7 walk. The position of complementarity of each ASO along the sequence of interest is indicated by a horizontal line. +: promotion of the exon 7 inclusion, −: inhibition of the exon 7 inclusion, *: no effect on alternative splicing. The figure is modified from refs [101,153] (copyright © 2021 Hua et al. [153]; © 2021 The American Society of Human Genetics [101]). The “ASO walking” experiment determined the splicing silencer elements (red underline).

ASOs act through antagonizing the binding between the pre-mRNA and trans-acting regulatory RNA elements or the formation of functional structures. ASO binding sequences are usually determined by an empirical method, “ASO walking”. Figure 9 illustrates two ASO walkings in the SMN2 gene within exon 7 [153] and intron 7 [101], respectively. 15-mer ASOs were used to target the region where neighboring ASOs overlapped by 10 nucleotides (exon 7 +16 to intron 7 +40 region is shown). In an in vitro splicing assay, ASOs masking exon 7 +36 to +50 and intron 7 +11 to +25 strongly induces the splicing of exon 7 [153]. Further optimization uncovered two ESS/ISS elements that can be targeted by ASOs: exon 7 +34 to +48, targeting TSL2 (Figure 9) [97], and intron 7 +11 to +24, an hnRNP A2/B1 binding site (Figure 9).

### 3.2. High-Throughput Screening (HTS) Assays for Small-Molecule Splicing Modifiers

The ASO-based splicing modifiers have some unfavorable pharmacokinetic properties. For example, they cannot cross the blood-brain barrier and have a low distribution in the bladder and stomach [55]. Small-molecule splicing modifiers have the propensity to overcome these problems. In addition, the identification of small-molecule splicing modifiers by phenotypic HTS assays enables an opportunity to uncover unknown pharmacological mechanisms. The mechanistic study of risdiplam analogs revealed that the drug unexpectedly increases the recruitment of FUBP 1 and displaces hnRNP G on the SMN2 pre-mRNA [28,109]. FUBP 1 had not previously been identified as an SMN2 splicing trans-acting regulatory protein prior to the mechanistic study. Interestingly, hnRNP G was previously shown as a splicing enhancer [154].

Phenotypic cell-based assays have been used to identify novel small-molecule splicing modifiers. In SMN2, exon 7 inclusion leads to a higher gene expression level, which can be further detected by immunostaining [156]. In an image-based HTS for full-length SMN2 inducer, fibroblasts from parental SMA carriers were seeded in a 384-well format and incubated with a chemical library [156]. The cells were fixed and treated using a standard immunostaining protocol with anti-SMN antibody and fluorescence secondary antibody [156]. The average intensity of SMN per cell was used as a criterium to identify primary hits [156].

Image-based HTS has also been used in reporter gene systems. In a screening for MAPT exon 10 splicing modifiers, a two-color splicing reporter was developed [157]. In this reporter system, a fusion green and red fluorescent proteins (GFP and RFP) was cloned to the downstream of the target exon, but the AUG start codon of GFP is split by MAPT exon 10 [157]. In this splicing reporter cassette, the GFP will not be expressed when MAPT exon 10 is included in the transcript, and RFP will be the only detectable fluorescent protein [157]. On the contrary, when MAPT exon 10 is skipped in the reporter transcript, the AUG start codon is rejoined, resulting in GFP expression [157]. In this case, the RFP expression is also reduced, because ribosomes initiate poorly at downstream open-reading frames. Therefore, in a screening performed in HEK293 cells, the green/red fluorescence intensity ratio was used to identify the hits [157]. Interestingly, in both screens for SMN2 and MAPT splicing modifiers, cardiotonic steroids were identified as a class of potent inducers exon inclusion [156,157].

**Figure 10:**
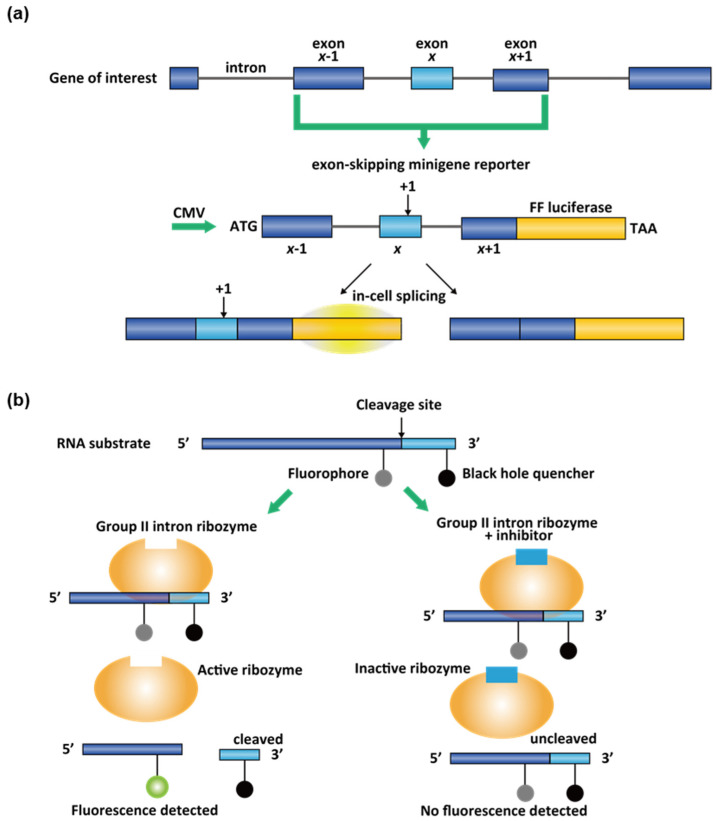
(**a**) Design of a minigene reporter assay for spliceosome-dependent splicing. By single-point addition, the firefly (FF) luciferase at the 3′-end will only be in-frame when exon *x* is included. (**b**) Design of in vitro FRET assay for group II intron splicing.

Cell-based luciferase minigene reporter with reading frameshifts is a sensitive assay to detect cellular alternative splicing change. In general, the target exon *x*, neighboring exons *x* − 1 and *x* + 1, and *cis*-acting splicing regulatory elements will be cloned into a vector flanked by a promoter and firefly (FF) luciferase reporter (Figure 10a) [158]. In the SMN2 splicing assay design, a single-point mutation makes the luciferase gene in-frame only if the exon 7 is included [159]. Both risdiplam and branaplam scaffolds were discovered by this luciferase reporter gene assays in HEK293 [155] and NSC34 motor neuron cells [108], respectively. For the counter-screen purpose, a complimentary assay can also be designed, where the luciferase gene is out-of-frame if the exon 7 is included [108]. This pair of reporter assays would eliminate most of the false positive hits. The luciferase assay design was also constructed for MAPT splicing [160].

Cell-free assays have also been used in phenotypic and target-based screenings. To identify yeast group II intron splicing inhibitors, an in vitro Förster resonance energy transfer (FRET) assays were used in screening in 384-well plates [10]. FRET signal is distance-dependent, and therefore, it reflects the interaction between the fluorescent donor–acceptor pairs in proximity [161]. The target self-splicing ai5γ group II intron is a ribozyme with complicated tertiary structures [10]. The catalytical domain can be extracted from the group II intron as a multiple-turnover ribozyme independent of the flanking exon sequences [10]. This simplified ribozyme can efficiently recognize and catalyze the cleavage of a designed exogenous RNA substrate oligonucleotide that contains the original 5′ splice site (Figure 10b) [10]. To monitor the splicing activity, a fluorophore and a fluorescence quencher were conjugated at positions on opposite sides of the cleavage site. Before the reaction, fluorescence is quenched because the fluorophore and the quencher on the same RNA strand are close in space. Once the RNA substrate was cleaved by the ribozyme, the fluorophore would be out of the interaction range with the quencher; in this way, fluorescence can be observed and measured to determine the splicing modulating activity of small molecules (Figure 10b) [10]. Notably, several polyphenolic compounds were identified as potent and well-behaved hits [10]. Therefore, it was suggested that these compounds bearing gallate or catechol moiety should not be simply filtered as pan-assay interference compounds (PAINS) [10,162].

**Figure 11:**
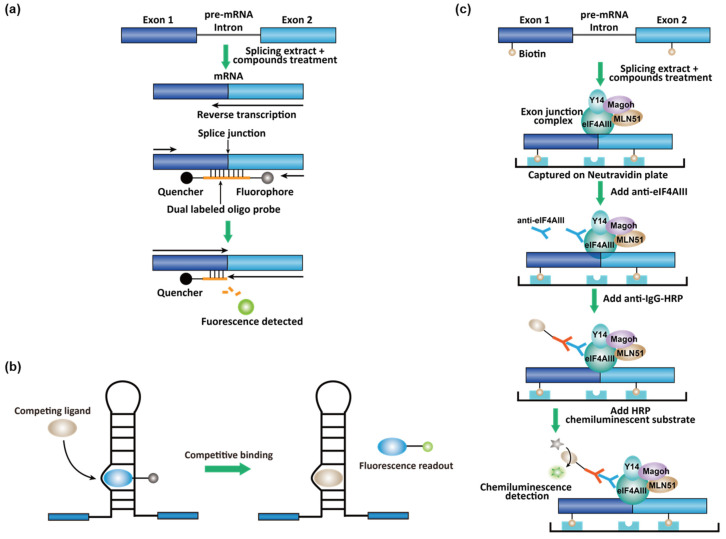
(**a**) RT-qPCR assay for the exon–exon junction sequence. (**b**) Fluorescent competition assay for MAPT exon 10 splicing. (**c**) Exon junction complex (EJC) detection assay (Copyright © 2021, American Society for Microbiology. All Rights Reserved [166]). HRP = horseradish peroxidase.

In vitro spliceosome-dependent assays using synthetic pre-mRNA and cell nuclear extract were also developed in an HTS format [163]. To identify spliceosome inhibitors, pre-mRNA substrate derived from the adenovirus major late transcript and HeLa nuclear extract were used in in vitro splicing reactions in 384-well plates [163]. The pre-mRNA was transcribed by T7 RNA polymerase and G(5′)ppp(5′)G-capped at the 5′-end. The reaction mixture was directly transferred into 384-well PCR plates for reverse transcription (RT)-quantitative PCR (qPCR) reactions [163]. A primer set and a Taqman probe were designed to target the exon–exon junction (Figure 11a). A Taqman probe is an oligonucleotide DNA that binds in the middle of the PCR amplicon, with a fluorophore–quencher FRET pair anchored at the two ends (Figure 11a). As the PCR reaction proceeds, the fluorophore is cleaved by the DNA polymerase and is no longer quenched. The fluorescence were, therefore, used as a readout to quantify the exon–exon junction (Figure 11a) [163]. Both natural product and small molecules were identified as modest spliceosome inhibitors (IC50 in the 20–50 μM range) at different stages in spliceosome assembly.

Affinity-based in vitro assays were also used in the high-throughput screening. To identify inhibitors for MAPT exon 10 splicing, a screening campaign was conducted for small-molecule stabilizers of the exon 10 stem-loop splicing silencer (see Section 2.5) using a fluorescent competition assay [144]. In this assay, pyrene-conjugated neomycin is a known intercalator of the exon 10 stem-loop and the fluorescence of the pyrene was reduced in the binding conformation. A competing ligand that releases the pyrene–neomycin restored the fluorescence, which was used as a readout (Figure 11b) [144]. Mitoxantrone was identified as a screening hit and was validated in splicing assays [144].

A hallmark of the completion of splicing is exon junction complexes (EJCs) ~20 nts upstream of the exon–exon junction deposited by spliceosomes in a sequence-independent manner [164,165]. EJCs control the nonsense-mediated decay and may play a role for exporting mRNAs from the nucleus into the cytosol [164,165]. Therefore, EJCs can be used as the biomarker for splicing events. To identify RNA splicing inhibitors of adenovirus type 2 construct with a deletion of intervening sequence (Ad2ΔIVS), a splicing-dependent EJC immunoprecipitation assay was developed [166]. A biotinylated UTP was spiked in the in vitro transcription reaction to produce the biotinylated pre-mRNA [166]. Biotinylated pre-mRNA was then incubated with nuclear extracts for splicing, and the spliced product was transferred into avidin-coated plates for immobilization. The EJC is quantified using primary antibodies to the EJC components Y14 and eukaryotic translation initiation factor 4aIII (eIF4A) and a horseradish peroxidase (HRP)-conjugated secondary antibody (Figure 11c) [166]. A small molecule that inhibits late-stage spliceosomes (i.e., after the first trans-esterification step) from this screen was identified.

## 4. The Chemical Space of RNA Splicing Modifiers

### 4.1. Chemical Modification of ASO

**Figure 12:**
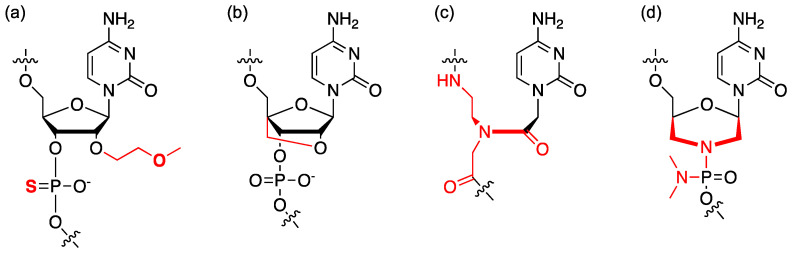
Chemical modifications of ASOs: (**a**) phosphorothioate and 2′-*O*-methoxyethyl (MOE), (**b**) locked nucleic acid (LNA), (**c**) peptide nucleic acid (PNA), and (**d**) phosphorodiamidate morpholino oligomer (PMO). Red = modifications to the native DNA or RNA structure.

The pharmacokinetic properties, stability, and target affinity of ASOs are controlled by the backbone and the chemical modification of the ribose (for review, see ref [55]). In cells and in vivo studies, the ASOs are usually modified extensively. In nusinersen, all the ribose units are 2′-alkylated by the *O*-methoxy-ethyl (MOE) group and all phosphate bridges are substituted with phosphorothioate (Figure 12a). DNA–RNA hybridization can trigger the RNase H activity, which specifically digests the RNA strand in a DNA-RNA duplex [50]. In modulating splicing, this RNase H activity is usually unwanted, and therefore, the native DNA backbone in ASO development for splicing modifiers should be avoided. In the RNA backbone, 2′-OH groups in the ribose account for the in-line cleavage of the backbone and some interactions with RNases, and therefore, 2′-MOE alkylation makes the ASO more stable (Figure 12a). The substitution of phosphorothioate provides the ASOs some additional resistance to RNases and increases the plasma protein binding and plasma stability [55]. A modified ribose, locked nucleic acid (LNA) (Figure 12b) was developed to “lock” the sugar puckering at the most favorable conformation for RNA duplex formation [167]. Incorporating LNA into ASOs significantly increases the melting temperature (*T*_m_ +3–8 °C per nucleotide [167]). LNA technology enables ASOs with higher sensitivity to single-point mismatches and allows a shorter possible ASO design with the same *T*_m_ [168].

ASOs with a DNA-/RNA-like backbone are densely negatively charged, resulting in high protein binding [55]. Neutral backbones were developed with the modifications of peptide nucleic acids (PNAs) (Figure 12c) and phosphorodiamidate morpholino (PMOs) (Figure 12d). PNA uses a branched *N*-(2-aminoethyl)-glycine to substitute ribose phosphate [169]; whereas PMO uses morpholine to substitute ribose and phosphorodiamidate to substitute phosphate. The non-ionic backbone of a PNA or PMO minimizes interactions with DNA/ RNA binding-proteins, eliminating potential non-antisense effects (e.g., innate immune response) [170]. Two PMO-based drugs have been approved by the FDA, eteplirsen and golodirsen. The cellular uptake mechanism of the ASOs is primarily endocytosis [50]. Conjugating the ASO with cell-penetrating peptide (CPP) is a promising approach to improve cellular uptake, and consequently making the ASOs more active [171,172]. The dimethyl amino moiety of the phosphoramidate in PMO can also be replaced by a piperazine, which is positively charged under physiological condition. And this positively charged PMO is termed PMOplus (for reviews in ASO drug development, see [173,174,175,176]).

### 4.2. Chemical Space of RNA-Targeting Small-Molecule Splicing Modifiers

Sequence-specific DNA- and RNA-binding small molecules have been sought after for decades for the use as therapeutic agents and chemical probes. For example, pyrrole-imidazole (Py-Im) polyamides can bind to the DNA minor groove, which is controlled by the side-by-side nucleobase pairs [177]. However, these DNA helix-binding molecules usually do not bind to RNA minor grooves efficiently [177], probably due to a larger minor groove in the A-form RNA helices [178]. A hallmark of RNA structure is the presence of complex secondary and tertiary structures through folding (for review, see [179]). Several secondary RNA structures were proposed to be recognizable, such as hairpins and internal bulges (for a recent review, see [180]). Some RNA–small molecule interaction databases have been built based on pattern recognition, including Inforna from the Disney group [181,182] and R-BIND from the Hargrove group [58]. It was suggested that RNA-binding small molecules share a “rod-like” shape based on an analysis of 104 reported RNA-binding molecules [58].

**Figure 13:**
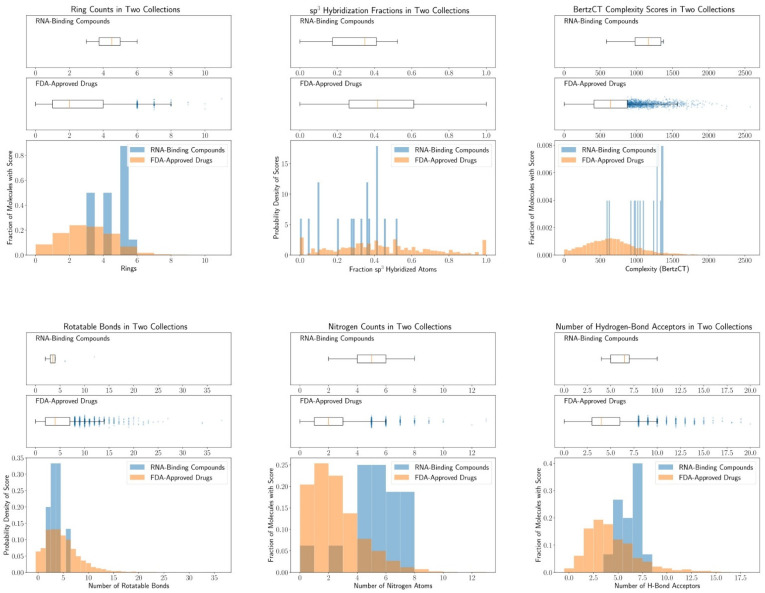
Distribution of key physicochemical properties of the known splicing modifiers and the FDA approved drugs in Table 4.

Here, we focused on small-molecule RNA splicing modifiers and summarized some key structural features from some known splicing modulators (Table 4, Figure 13, see Appendix A for cheminformatic analysis method). These splicing modifiers contain more hydrogen bonding acceptors, and specifically, more nitrogen atoms, than the average FDA-approved drugs (Table 4, Figure 13; see Table S1 for the list of FDA-approved drugs). At least one of the basic nitrogen atoms is protonated under physiological conditions in these known splicing modifiers and facilitates the interaction of the small molecule with the negatively charged RNA. We also found the small-molecule splicing modifiers are “flatter” than an average drug. The flatness is quantified by less rotatable bonds and more aromatic rings [183] (Table 4, Figure 13). Associated with the flatness, the ratio of sp^3^-hybridized carbon atoms to total sp^3^- and sp^2^-hybridized carbons (Csp3/[Csp2 + Csp3]) [184] is also reduced compared to the FDA-approved drugs (Table 4, Figure 13). A caveat for the drug development for these RNA-binding molecules is the potential selectivity and toxicity issues associated with the flatness [185]. Some risdiplam analogs were also documented for the induction of genome instability [65]. The off-target toxicity, however, can be circumvented through structure-activity-relationship studies [65]. The target identification of risdiplam analogs also uncovered that the small-molecule binding induces the association or dissociation of RNA-binding proteins [28,109]. The formation of the ternary structure of the RNA, small molecule, and RNA-binding protein will likely contribute to the high selectivity of risdiplam, in addition to the RNA sequence recognition [28,109]. Some other physicochemical properties do not significantly change in the known splicing modifiers, including the molecular weight, topological polar surface area (TPSA), and calculated Log*P* values. Information content of splicing modifiers calculated as BertzCT index is significantly higher than in FDA-approved drugs, the majority of which target proteins [186]. This is likely due to an increase in the number of rings and double bonds.

## 5. Conclusions

RNA splicing occurs in all kingdoms of life with distinct mechanisms and plays a pivotal role. In the recent two decades, the RNA-targeting splicing modifiers, including antisense oligonucleotides and small molecules, have become a new pharmacological modality for the treatment of human diseases or chemical probes for elucidating the molecular pathology. Currently, four ASO-based splicing modifiers were approved for DMD, while one ASO and one small-molecule drug were approved for SMA. Although the causes of DMD and SMA both tie to aberrant splicing, this pharmacological modality can be extended to other diseases, such as infectious diseases. In this review, we summarized the drug development targeting RNA splicing and known splicing modifiers. Phenotypic drug discovery, or forward pharmacology, is still a widely used approach to identify novel RNA splicing modifiers. However, with more understanding of the drug mechanisms, the target-based design also becomes realizable [9]. The chemical space for the RNA-targeting small-molecule splicing modifiers is different from the FDA-approved drugs. Bearing similar features to other RNA-binding molecules [58], the small-molecule splicing modifiers are flatter and possess more hydrogen bonding acceptors (particularly basic nitrogen atoms).

RNA-targeting approach drug discovery has its limitations. In some cases, although aberrant splicing is crucial for the pathology, RNA-targeting may not be an ideal approach for pharmacological intervention. For example, in the longest gene in the human genome, titin, the short pathogenic N2B isoform in cardiomyocytes has 168 exons skipped simultaneously from the long isoform N2BA [188]. It was demonstrated that a single trans-acting regulatory factor, RNA binding motif protein 20 (RBM20), controls the switching between the two splicing variants [189]. Some familial dilated cardiomyopathy is caused by a high N2B/N2BA ratio in cardiomyocytes [189]. Targeting cis-acting regulatory elements might be difficult to rescue inclusion of such a long RNA sequence in splicing. In some other cases, the conserved sequence of a splice site is destroyed by gene mutations. For example, a rare familial Parkinson’s disease is caused by c.1488+1G>A mutation in PTEN-induced kinase 1 (PINK1) gene. This G-to-A mutation occurs at the 5′-end of the intron 7 and makes the 5′ splice site of the exon 7 completely unrecognizable by the spliceosome. To correct the aberrant splicing, a gene-editing approach should be undertaken.

Despite the limitations, RNA-targeting splicing modifiers have already become an emerging pharmacological modality and enriched our chemical-genetic toolbox to modulate gene expression. Besides the four FDA-approved ASO drugs, the approval of risdiplam also underscored the possibility of a safe and sequence-specific RNA-targeting small molecule as a drug. Importantly, RNA-targeting is orthogonal to conventional protein-targeting strategies in drug development. For this reason, controlling gene isoform levels by splicing modifiers will be a promising approach to tackle the disease-modifying genes that are now considered “undruggable”.

## Figures and Tables

**Table 1:** Key differences between spliceosome-dependent, group I, group II intron, and tRNA splicing.

	Spliceosome	Group I Intron	Group II Intron	tRNA ^1^
**Organisms**	Eukaryotes	Bacteria, archaea, bacteriophages, plants, fungi	Fungi, algal plastids, bacteria, archaea	Eukaryotes, archaea
**Location**	Nucleus	Bacterial/archaeal, mitochondria, chloroplast	Bacterial/archaeal, mitochondria, chloroplast	vertebrate/plant nucleus, yeast cytosol, archaea
**Branch point**	A	Exogenous G cofactor	A	(not applicable)
**Intron size**	Dynamic, ~6.5 kb on average [46]	250–500 nt [39]	2–3 kb [47]	6–133 nt [48]
**Intron format**	Lariat	Linear	Lariat	Linear
**Protein involvement**	Spliceosome	Not necessary	Reverse transcriptase	Endonuclease, ligase

^1^ tRNAs in bacteria are sometimes spliced by the group I intron mechanism and are not included in this table.

**Table 2:** Disease-related splicing modifiers.

Disease	Target Gene	Name	Category ^1^	Development Stage
SMA	SMN2	Nusinersen [3]	ASO (MOE)	Approved
		E1^v1.11^-ASO [63]	ASO (PMO)	Phase I
		SMN-PNA [64]	ASO (PNA)	Cellular
		Risdiplam [65]	small molecule	Approved
		Branaplam [66]	small molecule	Phase II/III (discontinued)
		PK4C9 [67]	small molecule	Preclinical
		LDN-2014 [68]	small molecule	Preclinical
		HSMNEx7D [69]	ASO (PMO)	Preclinical
DMD	Dystrophin Ex51	Eteplirsen [70]	ASO (PMO)	Approved
	Dystrophin Ex51	Drisapersen [71]	ASO (2′-OMe)	Discontinued
	Dystrophin Ex53	Golodirsen [72]	ASO (PMO)	Approved
	Dystrophin Ex53	Viltolarsen [5]	ASO (PMO)	Approved
	Dystrophin Ex45	Casimersen [73]	ASO (PMO)	Phase III
	Dystrophin Ex45	DS-5141 [74]	ASO (ENA ^2^)	Phase I/II
Yeast infection	group II intron	Intronistat B [10]	small molecule	Cellular
FTDP-17	MAPT	E1.4, E5.3 [75]	ASO (PMO)	Preclinical
		E10α, E10β [76]	ASO (2′-OMe)	Cellular
		bipartite ASO [77]	ASO (PNA)	Cellular
		Mitoxantrone– bipartite ASO [78]	small molecule-ASO conjugate	Cellular
		compound 9 [9]	small molecule	Cellular
		compound 2 [79]	small molecule	Cellular
Familial Dysautonomia	IKBKAP	7-26S [11]	ASO (MOE)	Preclinical
		SSO1 [80]	ASO (OMe)	Cellular
		ExSpeU1 [81]	ASO	Preclinical
		Kinetin [82]	small molecule	Phase I (discontinued)
		RECTAS [83]	small molecule	Cellular
Influenza A virus infection	RNA segment 7	Radavirsen [8]	ASO (PMO)	Phase I

^1^ See Section 4.1 for abbreviations of ASO categories. ^2^ ENA = 2′-O, 4′-C-ethylene-bridged nucleic acid [84], an analog of locked nucleic acid (LNA).

**Table 3:** Selected human pathogenic viruses with essential RNA splicing processes.

Virus Name	Essential Genes	Viral Regulatory Factors
Influenza A virus	M1, M2/M42	NS1 [118]
human immunodeficiency virus 1	tat, rev; env	rev [128]
Human T-lymphotropic virus	env, tax, rex	rex [130,131]
Parvovirus B19	VP1, VP2	
Human Papillomavirus 1	E1, E2, L1, L2	E2 [132]

**Table 4:** Key physicochemical properties of the known splicing modifiers ^1^ and the FDA approved drugs (until 2008) ^2^.

Molecular Descriptor	RNA-Targeting Splicing Modifier	FDA-Approved Drugs
Ratio of sp^3^-hybridization	0.29 (±0.16)	0.44 (±0.26)
Rotatable bonds	4.1 (±2.4)	4.9 (±3.7)
Hydrogen bonding donor	2.3 (±2.3)	1.7 (±1.7)
Hydrogen bonding acceptor	6.4 (±1.5)	4.4 (±2.7)
Number of nitrogen atoms	4.9 (±2.0)	2.0 (±1.8)
Topological polar surface area (TPSA)	89 (±29)	73 (±49)
Number of rings	4.3 (±0.9)	2.6 (±1.5)
BertzCT	1113 (±253)	669 (±363)
Molecular weight	380 (±61)	332 (±126)
Calculated Log*P*	2.8 (±1.1)	2.5 (±2.3)

^1^ Include branaplam, risdiplam, SMN-C2, SMN-C3, SMN-C5, RG7800, PK4C9, TEC-1, mitoxantrone, intronistat A, intronistat B, kinetin, RECTAS, compound 9. ^2^ The physicochemical properties were calculated by RDKit [187]. The standard deviation values were included in the parentheses.

## Data Availability

The data presented in this study are available in Supplementary Materials.

## Supplementary Materials

The following are available online. Table S1: Simplified molecular-input line-entry system (SMILES) strings for all FDA-approved drugs used in Table 4 and Figure 13.

## Appendix A. Cheminformatic Analysis

We used the simplified molecular-input line-entry system (SMILES) representations of RNA-targeting splicing modifiers and of the FDA-approved drugs. The list of FDA-approved drugs was downloaded from https://drugcentral.org/ (accessed on 12 April 2021). It includes drugs approved as of September 2020. The comma-separated value tables containing SMILES strings for these compounds were then loaded into a Jupyter notebook running a Python 3.7 kernel with the Pandas library. RDKit [187] (https://www.rdkit.org/ accessed on 12 April 2021) was then used to convert SMILES into RDMol objects that were suitable for further analyses. To restrict the set of FDA-approved drugs to small molecules only, we set a cut-off of 750 Da above which a molecule was excluded from our analysis. This left us with approximately 95 % of the original molecules remaining.

For each molecule in their respective group, we used RDKit to calculate the ratio of sp^3^ hybridized carbons to all heavy atoms, the number of rotatable bonds, the number of hydrogen donors and acceptors, the number of nitrogen atoms, the topological polar surface area, the number of rings, the molecular weight, calculated partition coefficient (logP), and molecular complexity according to the topological index introduced by Bertz [186]. The modules for these calculations are used directly from RDKit without any additional modifications. With the values of descriptors per molecule, we then computed the mean and standard deviation for each descriptor within the two groups.
